# Novel Non-Hot Spot Modification in Fks1 of Candida auris Confers Echinocandin Resistance

**DOI:** 10.1128/aac.00423-23

**Published:** 2023-05-24

**Authors:** Milena Kordalewska, Geselle Cancino-Prado, João Nobrega de Almeida Júnior, Igor Brasil Brandão, Renata Tigulini de Souza Peral, Arnaldo L. Colombo, David S. Perlin

**Affiliations:** a Center for Discovery and Innovation, Hackensack Meridian Health, Nutley, New Jersey, USA; b Escola Paulista de Medicina, Federal University of São Paulo, São Paulo, Brazil; c Hospital Israelita Albert Einstein, São Paulo, Brazil; d Hospital da Bahia, Salvador, Brazil; e Brazilian Ministry of Health, Brasília, Brazil

**Keywords:** *Candida*, *Candida auris*, FKS1, anidulafungin, antifungal resistance, antifungal susceptibility testing, caspofungin, echinocandin resistance, echinocandins, micafungin

## Abstract

We determined the echinocandin susceptibility and *FKS1* genotypes of 13 clinical isolates of Candida auris that were recovered from 4 patients at a tertiary care center in Salvador, Brazil. Three isolates were categorized as echinocandin-resistant, and they harbored a novel *FKS1* mutation that led to an amino acid change W691L located downstream from hot spot 1. When introduced to echinocandin-susceptible C. auris strains by CRISPR/Cas9, Fks1 W691L induced elevated MIC values to all echinocandins (anidulafungin, 16 to 32×; caspofungin, >64×; micafungin, >64×).

## TEXT

Echinocandins are antifungal lipopeptides that noncompetitively inhibit β-1,3-d-glucan synthase, which catalyzes the synthesis of the 1,3-β-glucan polymers of the fungal cell wall. In *Candida* spp., pharmacodynamic resistance to echinocandin class drugs occurs predominantly when a fungal strain develops mutations in two highly conserved hot spot (HS) regions of the *FKS* genes that encode the catalytic subunits of β-1,3-d-glucan synthase. *In vitro*, the presence of amino acid substitutions in Fks subunits manifests as elevated MIC values (1).

Candida auris is an emerging fungal pathogen that exhibits elevated rates of antifungal drug resistance (including resistance to echinocandin class drugs) and causes hospital outbreaks (2). In C. auris, Fks1 HS regions are located at amino acid positions F635 to P643 (HS1), and D1350 to L1357 (HS2), and echinocandin resistance-conferring mutations were reported at several HS positions in clinical isolates (Table 1). Moreover, C. auris *FKS1* mutants harboring F635Δ (HS1) and M690I (downstream from HS1) were obtained through the serial *in vitro* caspofungin exposure of the strain B112020 (clade II, Japan, type strain) (3). Additionally, S639F, S639Y, D642Y, R1354H, and R1354Y *FKS1* mutants, in the NCPF 8971 strain background (clade I), were isolated after echinocandin treatment in a murine gastrointestinal colonization model (4).

**TABLE 1:** Echinocandin resistance-conferring alterations found in the Fks1 of Candida auris clinical isolates

Hot spot	Phenotype (Fks1)		Genotype (*FKS1*)	References
HS1	WT	F L T L S L R D P	TTCTTGACTTTGTCCTTGAGAGATCCT	Reference gene: B9J08_000964
	F635Δ	- L T L S L R D P	- - - TTGACTTTGTCCTTGAGAGATCCT	15
	F635Y	**Y** L T L S L R D P	T**A**CTTGACTTTGTCCTTGAGAGATCCT	16
	F635L	**L** L T L S L R D P	**C**^*a*^TCTTGACTTTGTCCTTGAGAGATCCT	16
	S639F	**F** L T L F L R D P	TTCTTGACTTTGT**T**CTTGAGAGATCCT	8, 15–17
	S639P	F L T L **P** L R D P	TTCTTGACTTTG**C**CCTTGAGAGATCCT	17 – 19
	S639T	F L T L **T** L R D P	TTCTTGACTTTG**A**CCTTGAGAGATCCT	20
	S639Y	F L T L **Y** L R D P	TTCTTGACTTTGT**A**CTTGAGAGATCCT	19, 21
	D642Y	F L T L S L R **Y** P	TTCTTGACTTTGTCCTTGAGA**T**ATCCT	17
HS2	WT	D W I R R Y T L	GACTGGATTAGACGTTATACCTTG	Reference gene: B9J08_000964
	R1354S	D W I R **S** Y T L	GACTGGATTAGA**A**GTTATACCTTG	16
	R1354H	D W I R **H** Y T L	GACTGGATTAGAC**A**TTATACCTTG	17

a Bold, modified amino acids or nucleotides; underlined, codons where modification occured.

Here, 13 isolates of C. auris (B61 to B73; clade I) that were recovered from 4 patients (A, *n* = 4; B, *n* = 7; C, *n* = 1; X, *n* = 1) at a tertiary care center in Salvador, Brazil, were evaluated for their echinocandin susceptibility and *FKS1* sequences. We performed antifungal susceptibility testing (AFST) with echinocandins (ANF, anidulafungin; CAS, caspofungin; MCF, micafungin), according to the CLSI methodology (5, 6), using C. parapsilosis ATCC 22019 and C. krusei ATCC 6258 as quality control strains. The MICs were interpreted by using tentative breakpoints, as suggested by the CDC (7). The full *FKS1* gene was amplified by PCR and sequenced by Sanger sequencing, and its sequence was analyzed (reference gene: B9J08_000964), as we described previously (8). 3 isolates that were recovered from the urine of patient A were categorized as echinocandin-resistant (ANF, 2 to 16 mg/L; CAS, >16 mg/L; MCF, >16 mg/L) and harbored a novel *FKS1* mutation, G2072T, which led to an amino acid change, namely, W691L (TGG → TTG). The remaining 10 isolates were echinocandin-susceptible (ANF MIC, 0.125 to 0.25 mg/L; CAS MIC, 0.125 to 0.25 mg/L; MCF MIC, 0.125 to 0.25 mg/L) and presented a WT *FKS1* genotype (Table 2). The full Fks1 sequences of clinical isolates B61 to B73 were deposited into the NCBI GenBank under the accession numbers OQ632632 to OQ632644.

**TABLE 2:** Antifungal drug susceptibility and Fks1 phenotypes of clinical isolates, parental strains, and transformants

Clinical isolate		Patient	Specimen	Fks1	GenBank accession number	MIC [mg/L]		
Study number	Strain code					ANF	CAS	MCF
B61	1923/2021	A	Urine	W691L	OQ632632	2	>16	>16
B62	1985/2021	A	Urine	W691L	OQ632633	16	>16	>16
B63	1854/2021	B	Urine	WT	OQ632634	0.125	0.125	0.125
B64	1858/2021	B	CVC tip	WT	OQ632635	0.125	0.125	0.125
B65	2075/2021	B	CVC tip	WT	OQ632636	0.25	0.25	0.25
B66	2076/2021	B	Blood	WT	OQ632637	0.25	0.25	0.25
B67	2077/2021	B	CVC tip	WT	OQ632638	0.125	0.125	0.125
B68	1537/2020	X	CVC tip	WT	OQ632639	0.125	0.125	0.125
B69	1982/2021	A	CVC tip	WT	OQ632640	0.25	0.25	0.125
B70	1980/2021	A	Urine	W691L	OQ632641	2	>16	>16
B71	1861/2021	C	NA	WT	OQ632642	0.25	0.25	0.25
B72	1921/2021	B	CVC tip	WT	OQ632643	0.125	0.125	0.125
B73	1922/2021	B	CVC tip	WT	OQ632644	0.125	0.125	0.125
DPL1384 (clinical isolate, clade I)				WT		0.125	0.5	0.25
DPL1384 + B61 *FKS1* (7 independent transformants)				W691L		2 to 4	>16	>16
CBS 10913 (type strain, clade II)				WT		0.06	0.25	0.125
CBS 10913 + B61 *FKS1* (3 independent transformants)				W691L		2	>16	>16

W691 in the Fks1 of C. auris is located 48 amino acids downstream from the *FKS1* HS1, and it is equivalent to W695 and W715 in the Fks1 of Saccharomyces cerevisiae and the Fks2 of C. glabrata, respectively (9, 10). According to Johnson and Edlind, the Fks1 W695 in *S. cereviasiae* is located in hot spot 3 (amino acids 690 to 700), which is predicted to be embedded within the outer leaflet of the membrane and may interact with the extended aliphatic tails of echinocandins (9). In C. glabrata, a W715L amino acid substitution in Fks2 was shown to lead to elevated echinocandin MIC values (10, 11).

To confirm that the Fks1 W691L variant confers echinocandin resistance in C. auris, a wild-type (WT) *FKS1* gene fragment in echinocandin-susceptible C. auris DPL1384 (clinical isolate, clade I) and CBS 10913 (type strain, clade II) was replaced with a 465 bp *FKS1* fragment (C1899 to C2364) harboring W691L (amplified from isolate B61) by using a CRISPR/Cas9 system, as described previously (12), except that the cells were made electrocompetent with a Frozen-EZ Yeast Transformation Kit (Zymo Research, Irvine, CA, USA) (13). The repair template was PCR-generated with Q5 High-Fidelity DNA polymerase (New England Biolabs, Ipswich, MA) and Cau_FKS1_CRISPR_F1 (5′-CTTCTTCTTGACTTTGTCCTTGAGAGATCCT ATTAGAAACTTGTCCACC-3′), and Cau_FKS1_CRISPR_R1 (5′-GGTGCTCTCAAAGTTCTCTTGCCCTCGATTTCAGAAGGAACCTGGTGATAC-3′) primers. The crRNA target sequence was CGATTTCAGAAGGAACCTGGTGG, and the Cau_FKS1_CRISPR_R1 primer introduced a modified PAM site (without an amino acid change) into the PCR product to prevent the Cas9 digestion of the repair template. The transformants were selected on YPD plates containing 4 mg/L MCF. We previously used a similar selection approach to introduce the non-hot spot modification E655K in the Fks2 of C. glabrata (13). The correctness of the transformation was validated by PCR and sequencing of the entire *FKS1* gene (8). After that, the echinocandin MIC values of the correct transformants were determined according to the CLSI methodology (5, 6). When introduced to echinocandin-susceptible strains, *FKS1* W691L induced elevated MIC values to all echinocandins (ANF, 16 to 32×; CAS, >64×; MCF, >64×) (Table 2).

The interactions between Fks1 enzyme variants and echinocandin drugs are not well-understood. However, a recent study presenting the cryo-electron microscopy structures of the Fks1 of Saccharomyces cerevisiae revealed active enzyme-membrane interactions and altered lipid arrangements around the mutated enzymes (14). Although limited in its scope (only two mutated Fks1 positions were analyzed) the results provide insights into possible binding sites for echinocandin drugs and improve our understanding of why certain enzyme regions (hot spots versus non-hot spots) have the propensity to impact echinocandin drug susceptibilities.

In conclusion, we report Fks1 W691L as a novel, echinocandin resistance-conferring, non-hot spot modification in C. auris. Our discovery emphasizes that, despite the significant progress that has been made in recent years in understanding echinocandin resistance in C. auris, the diversity of the *FKS1* mutations that contribute to C. auris echinocandin resistance may be underestimated. Ultimately, improving the knowledge of the resistance mechanism will help diagnose and treat C. auris-infected patients.
